# Associations between toenail arsenic concentration and dietary factors in a New Hampshire population

**DOI:** 10.1186/1475-2891-11-45

**Published:** 2012-06-29

**Authors:** Joann F Gruber, Margaret R Karagas, Diane Gilbert-Diamond, Pamela J Bagley, M Scot Zens, Vicki Sayarath, Tracy Punshon, J Steven Morris, Kathryn L Cottingham

**Affiliations:** 1 Department of Biological Sciences, Dartmouth College, Hanover, NH 03755, USA; 2 Section of Biostatistics and Epidemiology, Dartmouth Medical School, Hanover, NH 03755, USA; 3 Biomedical Libraries, Dartmouth College, Hanover, NH 03755, USA; 4 Research Reactor Center, University of Missouri and Harry S Truman Memorial Veterans Hospital, Columbia, MO 65211, USA

**Keywords:** Arsenic, Epidemiology, Biomarkers, Lipids, Fatty acids, Fish, One-carbon metabolism, Folate

## Abstract

**Background:**

Dietary factors such as folate, vitamin B12, protein, and methionine are important for the excretion of arsenic via one-carbon metabolism in undernourished populations exposed to high levels of arsenic via drinking water. However, the effects of dietary factors on toenail arsenic concentrations in well-nourished populations exposed to relatively low levels of water arsenic are unknown.

**Methods:**

As part of a population-based case–control study of skin and bladder cancer from the USA, we evaluated relationships between consumption of dietary factors and arsenic concentrations in toenail clippings. Consumption of each dietary factor was determined from a validated food frequency questionnaire. We used general linear models to examine the associations between toenail arsenic and each dietary factor, taking into account potentially confounding effects.

**Results:**

As expected, we found an inverse association between ln-transformed toenail arsenic and consumption of vitamin B12 (excluding supplements) and animal protein. Unexpectedly, there were also inverse associations with numerous dietary lipids (e.g., total fat, total animal fat, total vegetable fat, total monounsaturated fat, total polyunsaturated fat, and total saturated fat). Finally, increased toenail arsenic concentrations were associated with increased consumption of long chain n-3 fatty acids.

**Conclusion:**

In a relatively well-nourished population exposed to relatively low levels of arsenic via water, consumption of certain dietary lipids may decrease toenail arsenic concentration, while long chain n-3 fatty acids may increase toenail arsenic concentration, possibly due to their association with arsenolipids in fish tissue.

## Background

Exposure to arsenic has been established as a significant human health threat. Chronic exposure to high concentrations of arsenic can cause skin lesions, cancer, developmental toxicity, neurotoxicity, cardiovascular diseases, and other health effects [1,2]. Long-term exposure to lower concentrations of arsenic is also of concern [3-5].

Millions of people worldwide are at risk due to consumption of water contaminated with inorganic arsenic [6,7], and we are all exposed to arsenic via food [1]. For example, staple foods such as rice contain appreciable arsenic concentrations, especially of the more toxic, inorganic forms [1,8-13]. Seafood – including fish – is also high in arsenic, but in the less-toxic, organic forms [14]. Recent studies suggest that in the general population, which consumes relatively small concentrations of arsenic in drinking water, diet plays a dominant role in overall exposure [1,8-13].

However, foods also contain factors that are important for one-carbon metabolism, the process by which inorganic arsenic (iAs) is methylated [15,16] and removed from the body. The process of methylation has two steps: first, monomethylarsonic acid (MMA) is formed, and then dimethylarsinic acid (DMA). All three forms (iAs, MMA, and DMA) are excreted from the body [17-20], but DMA is the most prevalent form of arsenic in urine [21]. DMA is generally believed to be less toxic than iAs, while the trivalent form of the intermediate product MMA may be the most toxic [20]. In populations with nutritional deficiencies and high exposure to inorganic arsenic via drinking water, dietary factors important to complete one-carbon metabolism – including folic acid [18,22,23], vitamin B12 [24], protein [24-26], and methionine [24,25] – appear to facilitate the excretion of arsenic from the body.

Less is known about the impact of dietary factors on the concentrations of arsenic in biologic tissues, such as toenails, over periods of months to years. Arsenic that has not been methylated (i.e., iAs) has a high affinity for keratin, which is found in high concentrations in nails and hair [27]. As a result, iAs can accumulate in hair and nails [27,28], both of which have been used as biomarkers of chronic exposure [29]. For example, toenail arsenic concentrations have been used as a biomarker of exposure both via water and foods [30-32].

We investigated the associations between toenail arsenic concentration and dietary factors in a population-based study in New Hampshire, USA. Based on previous studies with short-term biomarkers (blood, urine), we hypothesized that the dietary factors important for one-carbon metabolism (including folate, vitamin B12, methionine, and protein) would be inversely associated with toenail arsenic concentration.

## Methods

### Study population

We analyzed existing data from population-based case–control studies of bladder and skin cancer conducted among 25 to 74 year old residents of New Hampshire who utilize private groundwater wells as their household water source [33]. Groundwater arsenic concentrations for this population vary spatially, from <0.0003 to 180 μg/L across the state [34,35], creating a natural gradient of exposure to inorganic arsenic via drinking water. The Committee for the Protection of Human Subjects (CPHS) of Dartmouth College approved study materials and protocols (current CPHS #10107 & #11697) and participants provided informed consent according to the approved protocol.

### Data collection

Details of the study design are provided elsewhere [33,36-39]. Briefly, participants were interviewed, usually in their home, to obtain information on sociodemographic factors (e.g., smoking history, drinking water source). To minimize potential reporting biases, interviewers were masked to the case–control status of participants and neither interviewers nor participants were aware of the original study hypotheses [39]. Eighty-five percent of cases and 70% of controls were interviewed during the bladder cancer study [40], and 82% of cases and 73% of controls were interviewed in the skin cancer study [41].

Beginning November 1999 for bladder cancer cases, and August 2000 for skin cancer cases and controls, we asked participants to complete a written, validated, semi-quantitative food frequency questionnaire (FFQ) covering the preceding 12 month period [42,43]. More than 75% responded to this request. The FFQ asked about the consumption of 121 different items from seven broad categories (dairy, fruits, vegetables, eggs and meat, breads, beverages, and baked goods), as well as dietary supplements (e.g., vitamins), during the past year. Frequency options ranged from never to six or more times a day. Using this instrument, nutrient composition of each food was calculated according to the published methods of Willett and colleagues [44]. Total consumption of each dietary factor was then summed across foods and converted to a daily consumption rate. For vitamins and minerals, we evaluated consumption solely from foods (“without supplements”) and consumption including dietary supplements (“with supplements”). All factors examined are listed in Additional File 1.

Finally, participants provided toenail clippings and a sample of household tap water for analysis of total arsenic concentration using previously established protocols [45,46]. Overall, over 90% of participants provided a toenail clipping sample. The majority of subjects (70%) provided toenail clippings within 14 days of filling out the FFQ; 91.5% provided clippings within one year of the FFQ. Further, the vast majority (93.5%) of toenail clippings were analyzed within 1 year of sample collection. Toenail clippings were analyzed at the University of Missouri Research Reactor Center using standard-comparator instrumental neutron activation analysis [47]. Nail samples were carefully washed prior to analysis to remove external contamination. Drinking water samples were analyzed at the Dartmouth Trace Element Analysis Core following the procedures described in Karagas et al. [45]. Specific quality control measures were taken to ensure the accuracy of laboratory results for both water and toenail samples [45].

### Statistical analyses

Prior to statistical analysis, we normalized data on toenail arsenic concentrations using a natural log transformation. We then excluded subjects who did not meet the caloric thresholds suggested by Willett [48]: 18 men below 800 calories and 13 above 4000 calories, and three women below 500 calories and four above 3500 calories. We also excluded one individual with an extremely high toenail arsenic concentration (7.626 μg/g), which was 420% higher than the next highest concentration.

We evaluated the relation between ln-transformed toenail arsenic and each dietary factor using general linear models (SAS version 9.2), focusing on those factors that had a statistically significant (α = 0.05) regression coefficient. For such factors, we confirmed that results were robust to outliers. We explored associations both for the whole population and stratified by arsenic concentration in the subjects’ household water supply (<1 μg/L versus ≥1 μg/L), since 1 μg/L is the concentration above which associations between toenail arsenic and drinking water arsenic concentrations typically emerge [39].

All analyses were conducted both with and without adjustment for potential confounding factors. We retained confounders that were deemed important from previous literature [45,49,50], biological plausibility, and univariate associations. We adjusted for five categorical variables (sex, smoking status [never/ever], season of toenail collection [winter, spring, summer, fall], case–control status [control, bladder cancer, basal cell carcinoma, squamous cell carcinoma], and body mass index (BMI) [normal (<25 kg/m^2^), overweight (between 25 kg/m^2^ and 30 kg/m^2^), obese (≥30 kg/m^2^), missing]) [51] and four continuous variables (age [years], daily intake of water from the household water source [L/d], total energy intake [kcal/d], and water arsenic concentrations for individuals with water arsenic concentrations ≥1 μg/L). Because seafood is a major source of organic arsenic [14], we also evaluated whether including total seafood consumption in our adjustment model affected our principal findings.

To account for multiple testing across many individual dietary factors, we used the false discovery rate (FDR) procedures implemented in the R package Q-value [52]. Specifically, we calculated the Q-value, the minimum FDR at which a test may be called statistically significant [53], from the combined list of P-values generated by our two major analyses (100 dietary factors each with a crude and adjusted model). Q-values ≤ 0.05 were considered statistically significant after correction for multiple testing.

Finally, to help interpret our regression coefficients, we determined the percent change in predicted, back-transformed toenail arsenic concentrations between first quartile and third quartile consumers for each dietary factor. We made predictions using our fully adjusted general linear models, focusing on non-smoking, control subjects with normal BMI whose toenails were collected during the most common season (fall) and whose water arsenic level was < 1 μg/L, which was the group containing the majority of participants. Using these categorical predictors, we then made separate predictions for males and females using the mean age, caloric consumption, and water consumption; median arsenic water concentration; and first and third quartile for the dietary factor. By using two separate predictions, we could confirm that patterns of change were generally robust to sex.

## Results and discussion

Our study population included 920 individuals with household water arsenic concentrations ranging from 0.004 μg/L to 158 μg/L (median 0.303), with a median toenail arsenic concentration of 0.085 μg/g (IQR, 0.059 – 0.132; range 0.016 – 1.816). Household water arsenic concentrations were generally quite low: 77% of the population had household water <1 μg/L and 94% had water <10 μg/L. For the 213 individuals exposed to ≥1 μg/L of arsenic in water, the median water arsenic concentration was still relatively low, 4.154 μg/L (IQR, 2.253 – 10.160; range 1.019 – 158.050). Additional characteristics of the study population are summarized in Table 1. Summary statistics for those dietary factors found to be significantly related to toenail arsenic are provided in Additional File 2.

**Table 1:** Population characteristics, including information about the variables included in the adjustment model (n = 920)

	**Total population (n = 920)**		**Population with water [As] < 1 μg/L (n = 707)**		**Population with water [As] between 1 and 10 μg/L (n = 157)**		**Population with water [As] > 10 μg/L (n = 56)**	
A. Continuous variables	**Mean ± 1SD**		**Mean ± 1SD**		**Mean ± 1SD**		**Mean ± 1SD**	
Toenail arsenic (μg/g)	0.122 ± 0.136		0.102 ± 0.096		0.149 ± 0.154		0.292 ± 0.297	
Water arsenic (μg/L)	2.711 ± 9.978		0.269 ± 0.225		3.806 ± 2.498		30.467 ± 27.942	
Age (years)	61.0 ± 10.0		61.1 ± 10.1		60.5 ± 9.9		61.7 ± 10.1	
Water consumption from household supply (L/d)	1.11 ± 0.77		1.11 ± 0.76		1.07 ± 0.75		1.17 ± 0.94	
Energy intake (kcal/d)	1919.1 ± 630.1		1907.3 ± 633.8		1934.8 ± 600.9		2024.6 ± 662.1	
B. Categorical variables	**n**	**%**	**n**	**%**	**n**	**%**	**n**	**%**
Sex								
Female	364	39.6	280	39.6	66	42.0	18	32.1
Male	556	60.4	427	60.4	91	58.0	38	67.9
Smoking status								
Never smoked	298	32.4	224	31.7	57	36.3	17	30.4
Has smoked	622	67.6	483	68.3	100	63.7	39	69.6
Season in which the toenail sample was collected								
Winter	186	20.2	142	20.1	30	19.1	14	25.0
Spring	182	19.8	144	20.4	32	20.4	6	10.7
Summer	268	29.1	191	27.0	52	33.1	25	44.6
Fall	284	30.9	230	32.5	43	27.4	11	19.6
Case–control Status								
Control	227	24.7	179	25.3	33	21.0	15	26.8
Bladder cancer	275	29.9	206	29.1	45	28.7	24	42.9
Basal cell skin cancer	214	23.3	162	22.9	40	25.5	12	21.4
Squamous cell skin cancer	204	22.2	160	22.6	39	24.8	5	8.9
Body mass index								
Normal (<25 kg/m²)	164	17.8	119	16.8	37	23.8	8	14.3
Overweight (≥25 kg/m² & <30 kg/m²)	176	19.1	142	20.1	28	17.8	6	10.7
Obese (≥30 kg/m²)	73	7.9	56	7.9	14	8.9	3	5.4
Missing	507	55.1	390	55.2	78	49.7	39	69.6

In preliminary analyses stratified by drinking water exposure (<1 μg/L vs. ≥1 μg/L), we did not find meaningful differences in the observed associations between toenail arsenic concentration and dietary factors. Association strength and direction were consistent between strata, but our ability to detect statistically significant associations increased with sample size, such that only the strongest associations were detected in the higher-exposure group (≥1 μg/L). As such, we report pooled analyses for the entire population below.

### Few factors associated with one-carbon metabolism were identified as significant predictors of toenail arsenic

Based on studies of urinary arsenic metabolites, we hypothesized that there would be negative relationships between toenail arsenic concentration and dietary factors known to be important for one-carbon metabolism (e.g., folic acid, protein, methionine, vitamin B12). However, we found that only animal protein and vitamin B12 without supplement had statistically significant, negative regression coefficients, and estimates of effects on toenail arsenic concentrations from Q1 to Q3 consumers were relatively small (<7%) (Table 2A). In addition, methionine was marginally significantly associated with toenail arsenic after adjustment for potential covariates (slope (CI) = -0.086 (-0.18, 0.0063); *P* = 0.068). These findings were robust to the inclusion of total seafood consumption in the adjustment model (Additional File 3).

**Table 2:** **Dietary factors that were significantly associated with toenail arsenic concentration (bold)**^**a**^: Q-values ≤ 0.05 are noted with a * next to the*P*value

**Variable**	**Regression slopes**					
	**Crude (n = 920)**		**Adjusted**^**a**^**(n = 914)**			
	***β*(95% CI)**	***P*value**	***β*(95% CI)**	***P*value**	**% change**^**b**^**Q1-Q3 males**	**% change**^**c**^**Q1-Q3 females**
**A.** Negative associations						
**Dietary lipids**						
Total fat (g)	-1.5E-03 (-3.0E-03, 3.5E-05)	0.056	**-5.8E-03 (-8.4E-03, -3.2E-03)**	<0.001*	**-20.6**	**-16.4**
Animal fat (g)	-1.8E-03 (-4.2E-03, 5.7E-04)	0.14	**-4.2E-03 (-7.2E-03, -1.2E-03)**	**0.006**	**-9.3**	**-7.1**
Vegetable fat (g)	-2.4E-03 (-5.1E-03, 3.0E-04)	0.081	**-4.9E-03 (-8.5E-03, -1.3E-03)**	**0.008**	**-10.6**	**-8.7**
Total unsaturated fat						
Total monounsaturated fat (g)	-3.6E-03 (-7.4E-03, 1.0E-04)	0.057	**-1.2E-02 (-1.8E-02, -5.6E-03)**	<0.001*	**-17.0**	**-14.1**
Palmitoleic fatty acid (g)	-4.4E-02 (-1.1E-01, 2.1E-02)	0.18	**-1.0E-01 (-1.8E-01, -1.9E-02)**	**0.016**	**-8.1**	**-6.8**
Oleic fatty acid (g)	**-4.2E-03 (-8.3E-03, -7.6E-05)**	**0.046**	**-1.3E-02 (-2.0E-02, -6.4E-03)**	<0.001*	**-17.1**	**-13.5**
Total polyunsaturated fat (g)	-5.5E-03 (-1.3E-02, 2.1E-03)	0.16	**-1.8E-02 (-2.9E-02, -7.3E-03)**	0.001*	**-13.2**	**-10.2**
Total n-6 fatty acids^d^ (g)	-6.1E-03 (-1.4E-02, 2.2E-03)	0.15	**-1.8E-02 (-2.9E-02, -6.5E-03)**	0.002*	**-11.7**	**-9.7**
Linoleic fatty acid (g)	-7.1E-03 (-1.6E-02, 1.6E-03)	0.11	**-2.0E-02 (-3.2E-02, -8.7E-03)**	<0.001*	**-13.0**	**-10.2**
Linolenic fatty acid (g)	-3.8E-02 (-1.2E-01, 4.1E-02)	0.35	**-1.6E-01 (-2.7E-01, -5.4E-02)**	0.003*	**-10.8**	**-9.3**
Arachadonic fatty acid (g)	-4.3E-01 (-1.1E + 00, 2.1E-01)	0.19	**-6.8E-01 (-1.3E + 00, -4.0E-02)**	**0.037**	**-5.3**	**-4.7**
Total saturated fat (g)	-3.5E-03 (-7.5E-03, 4.8E-04)	0.084	**-1.0E-02 (-1.6E-02, -4.5E-03)**	<0.001*	**-13.4**	**-10.8**
Lauric fatty acid (g)	**-1.2E-01 (-2.3E-01, -4.5E-04)**	**0.049**	**-1.7E-01 (-2.9E-01, -4.8E-02)**	**0.006**	**-6.5**	**-5.1**
Palmitic fatty acid (g)	-6.7E-03 (-1.4E-02, 8.2E-04)	0.081	**-2.2E-02 (-3.4E-02, -1.1E-02)**	<0.001*	**-15.5**	**-12.8**
Stearic fatty acid (g)	**-1.8E-02 (-3.3E-02, -3.0E-03)**	**0.018**	**-4.3E-02 (-6.5E-02, -2.1E-02**	<0.001*	**-15.6**	**-12.8**
Steroid						
Cholesterol (mg)	-2.9E-04 (-6.3E-04, 3.9E-05)	0.084	**-5.0E-04 (-8.7E-04, -1.4E-04)**	**0.007**	**-7.3**	**-6.2**
**Protein**						
Animal protein (g)	-7.3E-04 (-2.7E-03, 1.3E-03)	0.47	**-2.6E-03 (-5.0E-03, -1.5E-04)**	**0.037**	**-6.6**	**-6.4**
**Vitamins**						
Retinol^e^ (IU)	**-3.2E-05 (-5.7E-05, -7.3E-06)**	**0.011**	**-2.9E-05 (-5.2E-05, -6.1E-06)**	**0.013**	**-3.8**	**-3.2**
Vitamin B12^e^ (μg)	**-8.5E-03 (-1.6E-02, -1.1E-03)**	**0.024**	**-7.6E-03 (-1.5E-02, -5.2E-04)**	**0.035**	**-3.1**	**-2.3**
**Plant-compounds**						
Beta cryptoxanthin (μg)	-3.2E-04 (-6.6E-04, 2.9E-05)	0.072	**-3.7E-04 (-6.9E-04, -5.1E-05)**	**0.023**	**-6.1**	**-6.4**
**B.** Positive associations						
**Dietary lipids**						
Total unsaturated fat						
Polyunsaturated fat						
n-3 Fatty acids (EPA^f^,DPA^g^,DHA^h^) (g)		**0.035**	**1.4E-01 (4.6E-04, 2.8E-01)**	**0.049**	**3.5**	**3.4**
n-3 Fatty acids^i^ (EPA & DHA) (g)	**1.8E-01 (1.3E-02, 3.4E-01)**	**0.034**	**1.5E-01 (1.6E-03, 3.0E-01)**	**0.048**	**3.4**	**3.6**
EPA (g)	**4.7E-01 (9.2E-02, 8.4E-01)**	**0.015**	**4.0E-01 (5.9E-02, 7.4E-01)**	**0.022**	**3.7**	**3.7**
DPA (g)	**2.9E + 00 (4.4E-01, 5.3E + 00)**	**0.021**	2.2E + 00 (-6.7E-02, 4.4E + 00)	0.057	4.5	4.5
**Elements**						
Manganese^e^ (mg)	**3.9E-03 (6.8E-05, 7.7E-03)**	**0.046**	5.1E-03 (-2.0E-03, 1.2E-02)	0.16	9.9	9.6
**Alcohols**						
Ethanol (g)	**6.0E-03 (3.1E-03, 8.8E-03)**	<0.001*	**6.6E-03 (4.0E-03, 9.2E-03)**	<0.001*	**10.0**	**3.2**

^a^ Covariates included: sex, smoking status, season of toenail collection, case–control status, body mass index, age, daily intake of water from the household water source, total energy intake, and water arsenic concentrations for individuals with water arsenic concentrations ≥1 μg/L.

^b^ predictions made using: male, never-smoker, control, median water arsenic concentration for those <1 μg/L, normal BMI, fall toenail collection, and mean age, calories, and water consumption for males with water arsenic < 1 μg/L.

^c^ predictions made using: female, never-smoker, control, median water arsenic concentration for those <1 μg/L, normal BMI, fall toenail collection, and mean age, calories, and water consumption for females with water arsenic < 1 μg/L.

^d^ without Gamma-Linolenic Fatty Acid.

^e^ without supplements.

^f^ Eicosapentaenoic Fatty Acid.

^g^ Docosapentaenoic Fatty Acid.

^h^ Docosahexanenoic Fatty Acid.

^i^ without Alpha-Linolenic Fatty Acid.

One reason we may not have seen strong associations between toenail arsenic concentrations and the dietary factors involved in one-carbon metabolism is that our population had adequate intakes of these dietary factors. Nearly all subjects were at or above the Dietary Reference Intake (DRI) [54] for vitamin B12 (97.1%) and the majority was at or above the DRIs for total protein (87.2%) and folate (69.1%). This study was conducted after folic acid fortification of enriched grain products in the USA (phased in from 1996-1998 [55]), which likely helped contribute to adequate folate intake.

Alternatively, the factors affecting toenail arsenic concentrations, a long-term biomarker of arsenic exposure, may differ from the factors affecting short-term detoxification and excretion, as indicated by urinary arsenic concentrations. A study comparing long and short term biomarkers in both well-nourished and poorly nourished populations, with drinking water exposures ranging from very low (<1 μg/L) to >10 μg/L (the current USEPA drinking water standard) would help to identify which dietary factors have the greatest effect on toenail arsenic concentration.

### Many dietary lipids were inversely associated with toenail arsenic

**Figure 1:**
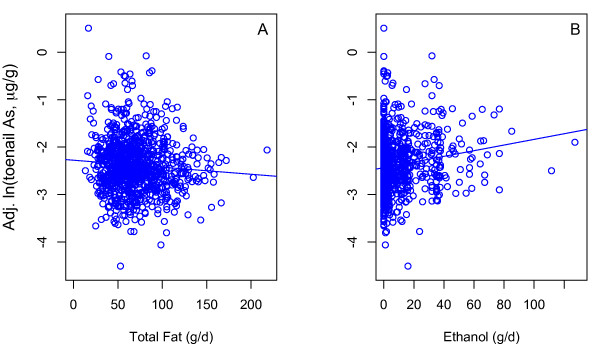
**Association of toenail arsenic concentration with total fat and alcohol in our case–control study population.** The adjusted, ln-transformed toenail arsenic concentration (mean plus the residuals from the adjustment model described in the text) varies inversely with (**A**) total fat consumption (g/d) and (**B**) positively with ethanol consumption (g/d). The blue line in each panel represents the least squares regression between the predictor and response variable, and so differs slightly from the lines fit in the full general linear model.

Of the 20 dietary factors inversely associated with ln-transformed toenail arsenic, 16 were dietary lipids (Table 2A) and these findings were robust to the inclusion of total seafood consumption in our adjustment model (Additional File 3). Ten of these factors remained significant after correction for multiple testing: total fat (Figure 1A), total monounsaturated fat, total polyunsaturated fat, total saturated fat, and several individual fatty acids (Table 2A). The magnitudes of effect varied, ranging from relatively small (4-8%) for several of the fatty acids to quite large (15-20%) for total fat (Table 2A).

It is not clear why so many dietary lipids were negatively associated with toenail arsenic in this study population. Until very recently, little research had been published on how dietary lipids affect arsenic metabolism; this past research shows either no effect or a detrimental effect of fats on the ability to methylate and excrete arsenic from the body (which presumably would result in a positive association with toenail arsenic concentrations). No mention is made of dietary lipids in many previous studies in humans [18,22-25], perhaps because they focused on poorly nourished populations in Bangladesh. Similarly, Steinmaus et al. [26] reported that fat, saturated fat, oleic fatty acid, linoleic fatty acid, and cholesterol consumption were unrelated to the proportions of urinary arsenic metabolites in a population in the western United States. In contrast, Basu et al. [56] found that animal fat intake was the strongest predictor of the percent MMA in urinary arsenic in a population in West Bengal, India. They hypothesize this finding is likely due to a reduction in the second step of methylation (MMA to DMA) rather than an increase in the first methylation step (iAs to MMA) [56]. Thus, our finding is novel.

From a physiological perspective it is conceivable that fat could form a complex with arsenic during digestion, inhibiting its absorption into the bloodstream and thus resulting in both decreased exposure and decreased toenail arsenic concentration. For example, lipid particles associated with proteins can interact with arsenic [57], which could provide an alternative mechanism for detoxification. Another possibility is that certain dietary lipids may have higher affinities for arsenic-containing compounds, preventing them from concentrating in toenails. Another plausible explanation is that foods with higher fat content contribute relatively less dietary arsenic compared to carbohydrate- and protein-rich foods (such as rice and fish) resulting in negative correlations in our energy adjusted analysis.

Further investigation is needed to determine whether there are biological mechanisms to account for why short-term and long-term biomarkers appear to produce different results regarding the effect of fats on arsenic biomarkers and whether the nutritional status of the individual plays a role in determining such different responses.

### Fatty acids typically found in fish oils were positively associated with toenail arsenic concentration

In contrast to the relationships with other dietary lipids, we found that intake of long chain n-3 polyunsaturated fatty acids, both individually and combined, were positively associated with toenail arsenic concentrations (Table 2B). Although magnitudes of effect were small (resulting in a 3-5% change in predicted toenail arsenic concentrations) and none of these variables was significantly associated with toenail arsenic after correction for multiple testing, we find these associations noteworthy. Eicosapentaenoic fatty acid (EPA) and docosahexaenoic fatty acid (DHA) are found in large quantities in fish oils [58,59] and docosapentaenoic fatty acid (DPA) is a biochemical intermediary between these forms [60]. Fish have some of the highest total arsenic concentrations in food [61,62]. This arsenic is expected to be predominantly in non-toxic organic forms such as arsenobetaine, which is not metabolized [50] and is excreted from the body relatively quickly [14]. However, fish also contain arsenolipids [63-66]; for example, cod-liver oil contains six arsenolipids immediately alongside long chain n-3 fatty acids [65]. Arsenolipids can be metabolized [64], which may release arsenic that can circulate and accumulate in toenails. We speculate that the positive associations between toenail arsenic concentrations and long chain n-3 fatty acids may be markers for a true underlying relationship between arsenolipids and toenail arsenic, a hypothesis supported by the fact that these associations were not detected when total seafood consumption was included in our adjustment model (Additional File 3). However, further studies are needed to confirm this relationship.

### Alcohol was positively associated with toenail arsenic

Consistent with previous epidemiological [32,67] and modeling [13] studies, we found strong positive associations between toenail arsenic concentrations and consumption of ethanol (Figure 1B, Table 2B), which remained significant after correction for multiple testing and after adjustment for total seafood consumption. This finding is also consistent with previous research on other biomarkers of arsenic exposure [68]. Among individuals exposed to varying concentrations of arsenic in drinking water, consumers of one or more alcoholic beverages per week had significantly higher proportions of inorganic urinary arsenic species when compared to individuals who consumed no alcoholic beverages [68]. This finding could be explained by alcohol’s ability to inhibit methionine synthase, which is important for arsenic metabolism [69]. Impeded metabolism of arsenic could also explain why toenail arsenic concentration was positively associated with alcohol consumption. In addition, alcoholic beverages such as beer and wine may themselves be a source of arsenic exposure [70], due to contamination of key ingredients.

### Miscellaneous dietary factors were associated with toenail arsenic

There were three dietary factors that had significant regression coefficients that did not remain significant after correction for multiple testing: retinol without supplements (negatively associated), beta cryptoxanthin (negatively associated), and manganese without supplements (positively associated). These relationships were likely a result of testing 100 dietary factors, since we know of no scientific reason that these dietary factors would be related to toenail arsenic.

### Potential limitations

Although toenails have limitations as biomarkers [29], these limitations likely did not affect our results. Orloff et al. (2009) identified three major problems with nails: (1) variability in growth rate, and thus the time period indicated by arsenic concentrations in nail clippings, (2) external contamination, and (3) inconsistent protocols for collection and analysis. Because our dietary information is averaged across a year, and our toenail clippings represent ca. 12 months of growth [71], we believe that the time scales for the predictor and response variables are matched appropriately. Contamination was minimized by having toenails collected immediately after bathing and scraping, and by sonicating nails in the laboratory prior to analysis [72]. Finally, all nails were analyzed in the same way, creating internal consistency within the study, even if we cannot make direct comparisons of toenail arsenic concentrations to other published studies [29].

Of greater relevance to the generality of our conclusions are the imprecision with which dietary factors were assessed and the inclusion of cancer patients among the study participants. First, our estimates of dietary factor consumption are likely to be less precise than our estimates of toenail arsenic concentration, violating one of the assumptions of general linear models. For example, recall bias could have affected the responses to the FFQ, potentially underestimating consumption of ‘unhealthy’ diet items (e.g., fatty foods) and overestimating consumption of ‘healthy’ items [73]. In addition, both the rate of intake of food items and the levels of dietary factors in foods can vary greatly over a one-year period, making it difficult to precisely estimate consumption of specific dietary factors. However, these errors would tend to bias results in favor of not detecting significant relationships, due to the so-called “errors in variables” problem [74]. In addition, while we adjusted for case status, many of the participants were cancer cases, which could affect the generalizability of our finding since it is plausible that these individuals process arsenic differently than non-cancer cases.

## Conclusion

This study tested the hypothesis that the dietary factors associated with toenail arsenic concentrations in well-nourished populations exposed to relatively low levels of water arsenic would be similar to the dietary factors associated with urinary arsenic concentrations in malnourished, highly exposed populations. Our findings do not support this hypothesis. Instead, several dietary lipids were inversely related to toenail arsenic concentration in our population, possibly because they inhibit the absorption of arsenic or its accumulation in toenails. However, long chain n-3 fatty acids (EPA, DPA, and DHA) were positively associated with toenail arsenic, potentially due to the association between these long-chain n-3 fatty acids and arsenolipids in fish tissue. Thus, our data suggest an association between dietary lipids and arsenic concentrations in biologic tissue.

## Competing interests

The authors declare that they have no competing interests.

## Authors’ contributions

JFG and KLC designed the research, conducted analyses, and drafted and critically revised the manuscript. MRK conceived and designed the study, obtained funding, collected and interpreted data, and critically revised the manuscript. DGD, PJB, and TP provided interpretations of results and critically revised the manuscript. MSZ collected data and conducted initial analyses, VS coordinated data collection and provided technical support, and JSM collected data and provided comments on the manuscript. All authors have read and approved the final manuscript.

## Supplementary Material

Additional file 1 **List of all dietary factors analyzed.** This table provides a list of all the dietary factors that were analyzed in this analysis. Click here for file

Additional file 2 **Quartiles of consumption of significant dietary factors.** This table provides summary statistics on the consumption of significant dietary factors. Click here for file

Additional file 3 **Dietary factors significantly associated (bold) with toenail arsenic with and without adjustment for seafood consumption.** This table provides a comparison of our presented results with results that also included adjustment for total seafood consumption. Click here for file
