# Supplementary material for: Associations between toenail arsenic concentration and dietary factors in a New Hampshire population
Source: Nutr J. 2012 Jun 29;11:45. doi: 10.1186/1475-2891-11-45 (PMC3426470; doi:10.1186/1475-2891-11-45)
Supplement: Additional file 1 — List of all dietary factors analyzed. This table provides a list of all the dietary factors that were analyzed in this analysis. [file 1475-2891-11-45-S1.pdf]

Additional File 1. List of all dietary factors analyzed.

Calories (kcal)

Sugars

- Carbohydrates (g)
- Sucrose (g)
- Fructose (g)
- Lactose (g)

Protein & Amino Acids

- Protein (g)
- Animal protein (g)
- Aspartate (g)
- Glutamate (g)
- Methionine (g)
- Tryptophan (g)

Dietary Lipids

- Total fat (g)
  - Animal fat (g)
  - Vegetable fat (g)
- Monounsaturated fat (g)
  - Palmitoleic Fatty Acid (g)
  - Oleic Fatty Acid (g)
  - Eicosenoic Fatty Acid (g)
- Polyunsaturated fat (g)
  - Linoleic Fatty Acid(g)
  - Linolenic Fatty Acid (g)
  - Arachadonic Fatty Acid (g)
  - Eicosapentaenoic Fatty Acid (EPA) (g)
  - Docosapentaenoic Fatty Acid (DPA) (g)
  - Docosahexaenoic Fatty Acid (DHA) (g)
  - n-3 Fatty Acids (EPA, DHA, DPA) (g)

Vitamins Continued

- Vitamin B2[a] (mg)
- Vitamin B3 (mg)
- Vitamin B3[a] (mg)
- Vitamin B5 (mg)
- Vitamin B5[a] (mg)
- Vitamin B6 (mg)
- Vitamin B6[a] (mg)
- Vitamin B12 (µg)
- Vitamin B12[a] (µg)
- Total Folate with supplements and fortified foods (µg)
- Total Folate[a] with fortified foods (µg)
- Natural Food Folate (µg)
- Folic Acid from supplements and fortified foods (µg)
- Folate Equivalents with supplements and fortified foods (µg)
- Vitamin C (mg)
- Vitamin C[a] (mg)
- Vitamin D (IU)
- Vitamin D[a] (IU)
- Total Vitamin E with supplements and fortified foods (mg)
- Vitamin E from food supplementation only synthetic (mg)
- Vitamin E mg[a] with fortified foods (µg)

Elements

- Calcium (mg)
- Calcium[a] (mg)
- Copper (mg)
- Copper[a] (mg)
- Iodine (µg)
- Iron (mg)

- n-3 Fatty Acids[a] (g)
- n-3 Fatty Acids (Alpha-Linolenic Fatty Acid, EPA, DHA, DPA) (g)
- n-3 Fatty Acids[a] (Alpha-Linolenic Fatty Acid, EPA, DHA, DPA) (g)
- n-3 Fatty Acids (EPA & DHA) no alpha-linolenic acid (g)
- n-6 Fatty Acids no gamma-linolenic acid (g)
- n-6 Fatty Acids[a] no gamma-linolenic acid (g)

Saturated fat (g)
- Butyric Fatty Acid (g)
- Caproic Fatty Acid (g)
- Caprylic Fatty Acid (g)
- Capric Fatty Acid (g)
- Lauric Fatty Acid (g)
- Myristic Fatty Acid (g)
- Palmitic Fatty Acid (g)
- Stearic Fatty Acid (g)

Cholesterol (mg)

Vitamins
- Vitamin A (IU)
- Vitamin A[a] (IU)
- Retinol (IU)
- Retinol [a] (IU)
- Retinol Equivalents of Vitamin A (µg)
- Retinol Equivalents of Vitamin A[a] (µg)
- Vitamin B1 (mg)
- Vitamin B1[a] (mg)
- Vitamin B2 (mg)

- Iron[a] (mg)
- Magnesium (mg)
- Magnesium[a] (mg)
- Manganese[a] (mg)
- Phosphorous (mg)
- Phosphorous[a] (mg)
- Potassium (mg)
- Potassium[a] (mg)
- Selenium (µg)
- Sodium (mg)
- Zinc (mg)
- Zinc[a] (mg)

Plant-Compounds
- Carotene (IU)
- Carotene[a] (IU)
- Beta Carotene (µg)
- Beta Carotene[a] (µg)
- Alpha Carotene (µg)
- Beta Cryptoxanthin (µg)
- Lycopene (µg)
- Lutein and Zeaxanthin (µg)

Miscellaneous
- Ethanol (g)
- Fiber (g)
- Caffeine (mg)

[a] without supplements
