# Supplementary material for: Associations between toenail arsenic concentration and dietary factors in a New Hampshire population
Source: Nutr J. 2012 Jun 29;11:45. doi: 10.1186/1475-2891-11-45 (PMC3426470; doi:10.1186/1475-2891-11-45)
Supplement: Additional file 2 — Quartiles of consumption of significant dietary factors. This table provides summary statistics on the consumption of significant dietary factors. [file 1475-2891-11-45-S2.pdf]

Additional File 2. Dietary factors significantly associated (bold) with toenail arsenic with and without adjustment for seafood consumption.

Variable	Adjusted[a] without Seafood (n=914)		Adjusted[a] with Seafood (n=877)	
	β	*P* value	β	*P* value
**Dietary Lipids**				
Total Fat (g)	**-5.8E-03**	**<.001**	**-5.8E-03**	**<.001**
Animal Fat (g)	**-4.2E-03**	**0.006**	**-4.6E-03**	**0.003**
Vegetable Fat (g)	**-4.9E-03**	**0.0082**	**-4.3E-03**	**0.024**
Total Unsaturated Fat				
Total Monounsaturated Fat (g)	**-1.2E-02**	**<.001**	**-1.2E-02**	**<.001**
Palmitoleic Fatty Acid (g)	**-1.0E-01**	**0.016**	**-1.2E-01**	**0.004**
Oleic Fatty Acid (g)	**-1.3E-02**	**<.001**	**-1.3E-02**	**<.001**
Total Polyunsaturated Fat (g)	**-1.8E-02**	**0.001**	**-1.9E-02**	**<.001**
Total n-6 Fatty Acids[b] (g)	**-1.8E-02**	**0.002**	**-1.7E-02**	**0.003**
Linoleic Fatty Acid (g)	**-2.0E-02**	**<.001**	**-2.1E-02**	**<.001**
Linolenic Fatty Acid (g)	**-1.6E-01**	**0.003**	**-1.9E-01**	**<.001**
Arachadonic Fatty Acid (g)	**-6.8E-01**	**0.037**	**-1.1E+00**	**0.003**
Total Saturated Fat (g)	**-1.0E-02**	**<.001**	**-1.0E-02**	**0.001**

Lauric Fatty Acid (g)	**-1.7E-01**	**0.006**	**-1.5E-01**	**0.018**
Palmitic Fatty Acid (g)	**-2.2E-02**	**<.001**	**-2.3E-02**	**<.001**
Stearic Fatty Acid (g)	**-4.3E-02**	**<.001**	**-4.3E-02**	**<.001**
Steroid				
Cholesterol (mg)	**-5.0E-04**	**0.007**	**-6.0E-04**	**0.002**
**Protein**				
Animal Protein (g)	**-2.6E-03**	**0.037**	**-3.8E-03**	**0.004**
**Vitamins**				
Retinolc (IU)	**-2.9E-05**	**0.013**	**-2.7E-05**	**0.029**
Vitamin B12[c] (µg)	**-7.6E-03**	**0.035**	**-9.3E-03**	**0.019**
**Plant-Compounds**				
Beta Cryptoxanthin (µg)	**-3.7E-04**	**0.023**	-2.7E-04	0.094
**Dietary Lipids**				
Total Unsaturated Fat				
Polyunsaturated Fat				
n-3 Fatty Acids (EPA[d],DPA[e],DHA[f]) (g)	**1.4E-01**	**0.049**	1.3E-01	0.25
n-3 Fatty Acids[g] (EPA & DHA) (g)	**1.5E-01**	**0.048**	1.4E-01	0.24
EPA (g)	**4.0E-01**	**0.022**	3.7E-01	0.11

DPA (g)	2.2E+00	0.057	1.6E+00	0.33
**Elements**				
Manganese[c] (mg)	5.1E-03	0.16	3.9E-03	0.3
**Alcohols**				
Ethanol (g)	**6.6E-03**	**<.001**	**6.5E-03**	**<.001**

[a] Covariates included: sex, smoking status, season of toenail collection, case-control status, body mass index, age, daily intake of water from the household water source, total energy intake, and water arsenic concentrations for individuals with water arsenic concentrations ≥1 µg/L.

[b] without Gamma-Linolenic Fatty Acid

[c] without supplements

[d] Eicosapentaenoic Fatty Acid

[e] Docosapentaenoic Fatty Acid

[f] Docosahexanenoic Fatty Acid

[g] without Alpha-Linolenic Fatty Acid
