# Supplementary material for: Associations between toenail arsenic concentration and dietary factors in a New Hampshire population
Source: Nutr J. 2012 Jun 29;11:45. doi: 10.1186/1475-2891-11-45 (PMC3426470; doi:10.1186/1475-2891-11-45)
Supplement: Additional file 3 — Dietary factors significantly associated (bold) with toenail arsenic with and without adjustment for seafood consumption. This table provides a comparison of our presented results with results that also included adjustment for total seafood consumption. [file 1475-2891-11-45-S3.pdf]

Additional File 3. Quartiles of consumption of significant dietary factors.

Dietary Factor	Minimum	Q1	Median	Q3	Maximum
Total Fat (g)	13.37	48.37	64.665	85.505	218.01
Animal Fat (g)	1.87	24.675	34.895	47.84	142.41
Vegetable Fat (g)	3.25	19.88	28.705	39.85	144.8
Total Monounsaturated Fat (g)	4.94	17.965	24.35	32.96	83.43
Palmitoleic Fatty Acid (g)	0.14	0.94	1.32	1.79	4.6
Oleic Fatty Acid (g)	4.47	16.28	22.015	29.455	77.85
Total Polyunsaturated Fat (g)	1.83	8.86	11.825	15.535	43.68
n-6 Fatty Acids no gamma-linolenic acid (g)	1.11	7.635	10.42	14.1	45.94
Linoleic Fatty Acid (g)	1.47	7.34	9.94	13.26	38.8
Linolenic Fatty Acid (g)	0.18	0.89	1.18	1.55	4.36
Arachadonic Fatty Acid (g)	0	0.09	0.13	0.17	0.49
Total Saturated Fat (g)	4.21	16.335	22.29	30.225	92.82
Lauric Fatty Acid (g)	0.07	0.3	0.44	0.65	2.93
Palmitic Fatty Acid (g)	2.42	9.205	12.46	16.59	47.2
Stearic Fatty Acid (g)	0.98	4.22	5.685	7.93	22.1
Cholesterol (mg)	17.67	183.77	249.175	324.63	1082.98
Animal Protein (g)	2.53	41.72	54.23	69	142.02
Retinol[a] (IU)	64.22	832.92	1365.685	2120.955	28778.68
Vitamin B12[a] (µg)	0.28	3.95	5.62	7.845	92.02
Beta Cryptoxanthin (µg)	0	49.115	124.32	221.205	1139.94
n-3 Fatty Acids (EPA[b], DHA[c], DPA[d]) (g)	0	0.11	0.235	0.36	2.49
n-3 Fatty Acids (EPA[b] & DHA[d]) no alpha-linolenic acid (g)	0	0.1	0.22	0.33	2.35
EPA[b] (g)	0	0.02	0.07	0.11	0.87
DPA[d] (g)	0	0.01	0.02	0.03	0.14
Manganese[a] (mg)	0.57	3.315	6.165	21.59	56.98
Ethanol (g)	0	0	1.79	12.16	127.18

[a] without supplements

[b] Eicosapentaenoic Fatty Acid

[c] Docosapentaenoic Fatty Acid

[d] Docosahexanenoic Fatty Acid
